# A Missing Piece in the Infrastructure to Promote Healthy Aging Programs: Education and Work Force Development

**DOI:** 10.3389/fpubh.2014.00287

**Published:** 2015-04-27

**Authors:** Janet Christine Frank

**Affiliations:** ^1^University of California at Los Angeles (UCLA) Center for Health Policy Research, The University of California at Los Angeles (UCLA) Fielding School of Public Health, Los Angeles, CA, USA

**Keywords:** gerontology education, career technical education, workforce development, competency-based education, faculty development, educational evaluation

There are compelling data available, both for the rapidly expanding older adult population, and for the value of evidence-based health promotion and disease management programs (EBHPs). The systems approach to transforming our aging services delivery system has been brilliant, but there is an important system missing – our educational system. Building the infrastructure to create embedded and accessible healthy aging programs must take into account workforce preparation. Most of the people currently working in the aging services delivery system are doing so without the benefit of any formal education or an organized course of study about older adults and aging services. For the state of California, 61% of aging services agencies reported zero current staff with formal gerontology education, defined as having had even one academic course in aging content (1). In a national study, less than half (46.6%) of responding Area Agencies on Aging (AAAs) had at least one staff with either a certificate or degree in gerontology and almost 27% have an Evidence-Based Program (EBP) Coordinator position (2, 3). There was no data reported on aging services workforce preparedness in program planning, implementation, and evaluation of EBHP, even for the EBP Coordinator positions.

There are several reasons, even with the availability of over 600 gerontology higher education programs nationwide, that our current aging services workforce lacks needed academic preparation. The first reason is a historical one: beginning employment in aging services may have pre-dated the widespread availability of gerontology education programs. Recent labor force studies have documented the “aging” of the aging services workforce, with impending mass retirement of long-time leaders and service providers. In fact, the California labor force study noted above documented that 52% of the aging services workforce is age 50 or over (1). And, the national study of aging services personnel echoed concerns about the “aging” of the aging services workforce, noting that about 20% of current staff is projected to retire within the next 5 years (by 2015) (2, 3). This means that workers nearing retirement age may have been entering college in the late 1960s and early 1970s. The first gerontology education programs at colleges began about 1972, and there were very few available until the 1980s. These anticipated high rates of retirement will soon lead to rapid turnover and the opportunity for new personnel replacements, perhaps with gerontology education backgrounds, at all levels. However, this “opportunity” assumes that current health and aging services leadership often without formal “aging” education will deem it a priority to hire available individuals with gerontology degrees or aging specialty education.

The second reason for aging services workforce preparation deficits is also historical. Workforce preparedness for our aging society has been an important topic for decades – beginning with the landmark publication of the U.S. Health Services Resource Administration, Bureau of Health Professions (HRSA BHPr), entitled, “A National Agenda for Geriatric Education: White Papers” (4). The National Agenda documented the lack of training and preparedness for the many needed health and social service professions and concerns for major service delivery systems and higher education. A number of important recommendations were made in this report. Unfortunately, slow and incremental progress has been achieved in addressing them, especially in the area of public health and aging. The documented gaps in preparedness from the 1995 report were resounded in the 2008 Institute of Medicine Report, “Retooling for An Aging America” (5). The 2008 IOM Report summarized critical workforce preparedness deficits and called for increased competencies in every type of health and social services personnel at every level.

The interesting thing is that geriatric and gerontologic competencies do exist for many health and social service disciplines, including medicine, nursing, social work, pharmacy, gerontology, and others (6–11). Public health currently does not have competencies specific to addressing the needs of older adults (12).

In spite of the call for action, the existence of professional competencies, and the estimated 600 current gerontology programs in higher education, are we graduating a sufficient number of people to fill positions vacated by retirement? The answer is no. We are losing ground, and we did not have much “ground” to lose. A recent article in the Chronicles of Higher Education discussed an 11% reduction in the number of gerontology degree programs between 2000 and 2010 (13). The reasons cited were low enrollments, budget cuts for higher education programs, and few student incentives, such as availability of scholarships. It is clear that reductions in state budgets for higher education, and lack of funding at the federal level, have taken a toll on gerontology education at the very time the programs should be robust and productive.

The Eldercare Workforce Alliance has documented the geriatric workforce shortfall (14). Simply stated, there are not enough people specializing in geriatrics and gerontology education to provide optimal care and services to the impending “boom” of older adults. This is primarily because outside of the degree and certificate specializations in gerontology, there are few courses offered. In addition, courses offered are typically elective, not required. For example, in 2009–2010, only 2.8% of BSW graduates and 6.7% of MSW graduates completed a specialization in aging. This is an average of 5% across all social work graduates (15). In accredited Schools and Programs in Public Health, the numbers are even lower. National data for the academic year 2004–2005 show that less than 3% of public health students enrolled in even one aging-related course (16).

How can the educational system be engaged in national systems change efforts to promote healthy aging and provide services to those most in need? First, there is a need to bring them to the table. National and state policy and planning meetings must include representatives from higher education systems (e.g., community college system) and professional schools (e.g., public health). They have been left out; and as a result, the exciting promise of EBHPs is a well-kept secret from academic programs. This new content is upbeat, engaging, and a perfect way to entice students to enroll in their first aging-related class. In addition, involving educational systems’ leadership in policy and planning meetings for systematic expansion of healthy aging programs will enhance the administrative support for aging related classes and programs at all levels of higher education and professional training. The “national movement” for healthy aging programs will be seen as an opportunity for increased demand in gerontology and geriatric education and training.

The national and state inclusion of systems level higher education leaders in policy discussions is needed. In addition, efforts to build relationships at the local level between healthy aging program providers with colleges and universities must be strengthened. Currently, only about 60% of AAAs have an established relationship with a local college or university for the purpose of securing well-trained personnel as positions become available (2, 3). Involving students early on in practical training through internships at agencies can lead to a pipeline of well-prepared graduates. These graduates are then available for employment as jobs open up – this is a win-win for all concerned. National and state conferences for aging services providers should include sessions on success stories and best practices in establishing and managing such local network opportunities. By showcasing successful models, perhaps we can move from 60% to more than 90% of agencies working with higher education in this manner.

The provision of student incentives is a second key activity to promote geriatrics and gerontology education to support the healthy aging movement. Incentives can be the type of traditional training grants with payment for tuition and student stipends that long ago were a component of the Older Americans Act. Incentives may also be less tangible, in the form of better branding of gerontology education as a central support for sustaining the healthy aging workforce. Increasing the number, strength, and purpose of collaborative relationships between educational institutions and aging services organizations is necessary. If aging services organizations could provide meaningful (and perhaps paid) internships for students to gain practical experience in healthy aging programs, this would definitely incentivize students to enroll into classes.

A third way to strengthen educational system involvement into the healthy aging movement is to assure the relevance of educational programs by developing new tailored curricula for EBHP and healthy aging. An established and tested model is the Skills for Healthy Aging Resources and Programs (SHARP) Career Technical Education Certificate Program. SHARP^©^ was developed in 2009 with funding from the U.S. Department of Education’s Funds for Improving Post-Secondary Education (FIPSE). It is a curriculum package that includes four first-year undergraduate courses. SHARP can be delivered as a component of community college or undergraduate programs, or as a stand-alone program for professional development of current aging services employees. It is competency-based and tailored to deliver content on healthy aging, behavior change, EBHP program implementation, and management. It also involves a service learning internship course that places students into agencies that are doing EBHPs.

SHARP has been delivered a total of six times in two California community colleges, with impressive evaluation results and much higher than average college retention (17). As a tested model educational program, it has been packaged for replication (curriculum, manual of procedures, evaluation tools, faculty development), so it can be adopted at other higher education institutions. A number of aging services providers have completed SHARP and brought its resources back into their agencies. Graduates of SHARP have been hired into agency positions, and agencies have even begun offering EBHP because of available SHARP graduates. Further information about SHARP can be requested from the author.

The education system may move slowly, but it can be responsive to workforce imperatives and addressing societal needs and opportunities. As the national movement for EBHP expansion and systems development was underway, the national education system was virtually ignored as a resource. Readying current agency personnel and recruiting volunteers to manage and lead EBHP was the focus of infrastructure capacity building to support programs. This may have been, by necessity, the first priority. However, to truly create national delivery systems and embed healthy aging programs into the fabric of how agencies and healthcare systems do their work, a steady supply of well-trained personnel are needed.

Looking to the future, it is imperative that content in gerontology, including EBHPs, is readily available within all levels of higher education programs. Utilizing social marketing principles to “brand” healthy aging curricula as essential and appealing may increase enrollments of students in a variety of disciplines. I can envision a future where all students graduating from any relevant program (health professionals, gerontology, public health, social services, business, public administration, etc.) are required to have coursework in healthy aging and EBHPs – it is just that important.

## Conflict of Interest Statement

The University of California at Los Angeles received grant funding from the U.S. Department of Education for the Skills for Healthy Aging Resources and Programs (SHARP) project from 2010 to 2013; SHARP is copyrighted by the University of California Regents.

This paper is included in the Research Topic, “Evidence-Based Programming for Older Adults.” This Research Topic received partial funding from multiple government and private organizations/agencies; however, the views, findings, and conclusions in these articles are those of the authors and do not necessarily represent the official position of these organizations/agencies. All papers published in the Research Topic received peer review from members of the Frontiers in Public Health (Public Health Education and Promotion section) panel of Review Editors. Because this Research Topic represents work closely associated with a nationwide evidence-based movement in the US, many of the authors and/or Review Editors may have worked together previously in some fashion. Review Editors were purposively selected based on their expertise with evaluation and/or evidence-based programming for older adults. Review Editors were independent of named authors on any given article published in this volume.
